# Development of KCC2 therapeutics to treat neurological disorders

**DOI:** 10.3389/fnmol.2024.1503070

**Published:** 2024-12-10

**Authors:** Shilpa D. Kadam, Shane V. Hegarty

**Affiliations:** Axonis Therapeutics Inc., Boston, MA, United States

**Keywords:** potassium chloride cotransporter 2 (KCC2), SLC12A5, neuronal inhibition, neurological disorders, CNS therapeutics

## Abstract

KCC2 is CNS neuron-specific chloride extruder, essential for the establishment and maintenance of the transmembrane chloride gradient, thereby enabling synaptic inhibition within the CNS. Herein, we highlight KCC2 hypofunction as a fundamental and conserved pathology contributing to neuronal circuit excitation/inhibition (E/I) imbalances that underly epilepsies, chronic pain, neuro-developmental/-traumatic/-degenerative/-psychiatric disorders. Indeed, downstream of both acquired and genetic factors, multiple pathologies (e.g., hyperexcitability and inflammation) converge to impair KCC2-dependent inhibition in CNS. When KCC2 hypofunction occurs, affected neurons are disinhibited due to impaired inhibitory responses to GABA/glycine. This causes neuronal hyperexcitability, disinhibition within neuron circuits, and disrupted neurological functions. More recently, KCC2 was identified as a genetically-validated target for epilepsy, intellectual disability, and autism spectrum disorder, and pathogenic mutations in human SLC12A5 gene were linked to psychiatric/mood disorders. The broad therapeutic utility of KCC2-upmodulating drugs relates to its critical role in determining inhibitory activity of GABAergic neurotransmission, a mechanism widely targeted by several drugs. However, in cases of KCC2 hypofunction GABAergic neurotransmission can be depolarizing/excitatory, thereby impairing endogenous neuronal inhibition while also limiting the effectiveness of existing therapeutics targeting/requiring GABAergic pathway inhibition. Several preclinical reports have shown that KCC2 upmodulating treatments rescue and increase the efficacy of anti-seizure and analgesic medications. Thus, a first-in-class KCC2-potentiating therapy would provide a novel mechanism for restoring physiological CNS inhibition and addressing drug resistance in patients with E/I imbalance pathologies. Herein, we discuss progress toward and further work needed to develop the first-in-class KCC2 therapeutics to treat neurological disorder patients.

## Introduction

The KCC2 transporter is the CNS neuron-specific Cl^–^ extruder essential for the establishment and maintenance of the transmembrane Cl^–^ gradient of mature neurons, thereby enabling synaptic inhibition within the CNS throughout life (Kaila et al., 2014). Inhibitory GABA/glycine signaling through ligand-gated chloride channels (e.g., GABA_*A*_Rs) depends on the Cl^–^ gradient across neuronal membranes, and the functional switch of GABA from excitatory-to-inhibitory during development is primarily mediated by the electroneutral KCC2 transporter (Kaila et al., 2014). Not only is Cl^–^ gradient required for inhibitory synaptic inhibition in the developing/mature CNS, it is also a primary mechanism regulating neuronal excitability (Pressey et al., 2023).

Since being characterized as the chief Cl^–^ extruder in CNS neurons, KCC2 has become a validated therapeutic target in recent years for several neurological disorders involving E/I imbalance within neuronal circuits. Significant KCC2-focused clinical and preclinical research efforts by clinicians and scientists over the past decade have implicated neuronal KCC2 hypofunction as a conserved pathology in epilepsies, chronic pain, neurodevelopmental, neurotraumatic, neurodegenerative, age-related, and psychiatric/mood disorders (Kaila et al., 2014; Duy et al., 2020; Tang, 2020; Belperio et al., 2022; Hegarty and Stanicka, 2022; Liedtke, 2022; Keramidis et al., 2023; Khademullah et al., 2023; Lam et al., 2023; Mcmoneagle et al., 2023; Pressey et al., 2023; Tomita et al., 2023; Mcardle et al., 2024). Indeed, downstream of both acquired and genetic factors, multiple pathological mechanisms (e.g., hyperexcitability, neuroinflammation, neurotrauma, impaired/delayed CNS maturation) converge to cause KCC2 hypofunction in neurons which impairs neuronal inhibition and leads to hyperexcitability in the nervous system (Rivera et al., 2002; Coull et al., 2003; Lee et al., 2011; Shimizu-Okabe et al., 2011; Puskarjov et al., 2012; Talos et al., 2012; Zhou et al., 2012; Duarte et al., 2013; Gagnon et al., 2013; He et al., 2014; Deidda et al., 2015; Tang et al., 2016; Dedek et al., 2019; Fukuda and Watanabe, 2019; Hinz et al., 2019; Tang et al., 2019; Verma et al., 2022; Sosunov et al., 2024). Affected CNS neurons are disinhibited by KCC2 hypofunction due to loss of inhibitory responses to GABA and glycine resulting in neuronal hyperexcitability and dysfunction within neuronal circuits. This leads to disruption of associated neurological functions resulting in a broad range of neuropathologies depending on which circuits are affected (e.g., seizures, pain, spasticity) (Woo et al., 2002; Coull et al., 2003; Boulenguez et al., 2010; Gagnon et al., 2013; Kaila et al., 2014; Fukuda and Watanabe, 2019). Despite this progress, there are no FDA-approved drugs that specifically potentiate or activate KCC2. In this perspective, we discuss efforts to develop first-in-class KCC2 therapeutics, highlight some key insights gained from recent literature to provide a practical roadmap for therapeutically targeting KCC2 to treat neurological disorders, and discuss the translational considerations for advancing first- and best-in-class KCC2 potentiators into the clinic to address unmet medical needs of a variety of neurological disorder patients are discussed.

## Preclinical development of KCC2 therapeutics

The broad therapeutic utility of KCC2 potentiator drugs relates to its critical role in determining the strength and inhibitory activity of GABA-/glycine-ergic neurotransmission, and thus its unique ability to restore synaptic inhibition to address pathological E/I imbalance within CNS circuits. Increasing inhibitory GABAergic neurotransmission is a validated therapeutic approach for many neurological disorders, with several approved CNS drugs targeting this pathway on the market and in clinical testing (Richardson et al., 2024). However, GABA_*A*_R agonists and positive allosteric modulators (PAMs) can cause global CNS activity depression by cell autonomously hyperpolarizing the resting membrane potential (RMP) of neurons, leading to dose-dependent cognitive impairment and sedation/somnolence/ataxia side effects which limit their therapeutic utility especially chronically. Additionally, in such cases medication tolerance is an important issue when patients need incrementally larger doses over time to achieve the same results. For example, benzodiazepines are well documented to develop tolerance after an initial “honeymoon” period of good seizure control in epilepsy, and therefore have a limited role in the long-term seizure management (Woldman et al., 2019). Activation/potentiation of the electroneutral KCC2 transporter hyperpolarizes the chloride equilibrium potential (ECl) to its homeostatic state in mature neurons, without affecting RMP, priming CNS neurons to be inhibited by endogenous GABA/glycine neurotransmission within inhibitory circuits, avoiding excessive CNS-wide depression of neuronal activity. The ability to fine-tune the neuronal Cl- transmembrane gradient to enable/restore physiological levels of inhibitory neurotransmission at GABA-/glycine-ergic synapses is a therapeutic profile unique to KCC2 potentiators, as KCC2 is the only transporter than can extrude Cl^–^ from CNS neurons to set/reset the ECl. Indeed, inhibition of Cl^–^ import is not sufficient to achieve Cl^–^ extrusion, which likely underlies why KCC2 knockout mice have lethal generalized seizures postnatally at a time when KCC2 function is required to establish inhibitory neurotransmission in the CNS (Woo et al., 2002; Kaila et al., 2014). Moreover, despite several approved therapies, an unmet need for many patients experiencing epilepsy, chronic pain, and other CNS disorders is refractoriness or non-responsiveness to first-line medications, whose doses are also limited by their tolerability issues. Recurrent excitation in pathological CNS circuits drives KCC2 hypofunction and consequently GABAergic neurotransmission can become depolarizing/excitatory, thereby impairing neuronal inhibition while also limiting the effectiveness of existing therapeutics targeting or requiring GABAergic pathway inhibition for efficacy. Therefore, KCC2 hypofunction has been implicated in drug-resistance mechanisms, and increasing/restoring KCC2 function/expression has been shown to rescue the efficacy, and/or increase the therapeutic window, of first-line anti-seizure and analgesic medications (Li et al., 2016; Ferrini et al., 2017; Li et al., 2020; Lorenzo et al., 2020; Sullivan et al., 2021; Aby et al., 2022; Cheung et al., 2022; Lee et al., 2022; Paige et al., 2022; Jarvis et al., 2023; Shi et al., 2023). Indeed, KCC2-upmodulating treatments rescue the efficacy of first-line anti-seizure medications (ASMs), such as benzodiazepines and valproic acid, as well as the analgesic efficacy of selective serotonin reuptake inhibitors, morphine and NMDAR antagonist mechanisms in animal models (Li et al., 2016; Ferrini et al., 2017; Li et al., 2020; Lorenzo et al., 2020; Sullivan et al., 2021; Aby et al., 2022; Cheung et al., 2022; Lee et al., 2022; Paige et al., 2022; Jarvis et al., 2023; Shi et al., 2023).

Tremendous preclinical progress has been made toward the development of first-in-class drugs that specifically potentiate/activate KCC2 in recent years. The CNS neuron-specific expression profile of KCC2 makes it a very attractive target for a CNS-penetrant small molecule, without the risk of on-target toxicity in peripheral tissues/organ systems or even other non-CNS neuronal cell types of the nervous system. Firstly, Yves De Koninck’s lab and Chlorion Pharma screened over 92,500 drug-like molecules to identify KCC2 enhancers in immature neuronal-like cells endogenously-expressing low levels of KCC2, designed to recapitulate KCC2 hypofunction pathology and to facilitate detection of increased KCC2 activity. This Cl^–^ extrusion (Clomeleon sensor) screen led to the development of CLP257, its prodrug CLP290, and other independent hit compounds as putative KCC2 functional enhancers that are capable of increasing KCC2-dependent Cl^–^ extrusion, KCC2 membrane expression, and hyperpolarizing the ECl (E_*GABA*_) in neurons (Gagnon et al., 2013). Importantly, Gagnon et al. (2013) also showed that the oral efficacy for analgesia of CLP290 in a neuropathic pain model was similar to the current first-line analgesic pregabalin, but critically without its sedation side effects. Since then, several groups have utilized CLP257/CLP290 to demonstrate how these KCC2 potentiator tool compounds can acutely and chronically treat epilepsy, pain, spasticity, neurodegeneration and neurotrauma in various animal models as well as patient tissues, demonstrating the broad therapeutic promise of this KCC2 potentiation approach (Ferrini et al., 2017; Chen et al., 2018; Lorenzo et al., 2020; Bilchak et al., 2021; Dzhala and Staley, 2021; Sullivan et al., 2021; Aby et al., 2022; Paige et al., 2022; Bilchak et al., 2023; Donneger et al., 2023; Keramidis et al., 2023; Khademullah et al., 2023; Pan et al., 2024).

More recently, the Moss lab and AstraZeneca screened 1.3 million compounds for activation of overexpressed KCC2 in HEK-293 cells (Jarvis et al., 2023). This thallium (Tl^+^) influx screen identified the Ovid Therapeutics licensed compound, OV350, which binds to and activates KCC2 without modifying its plasma membrane expression or some regulatory phosphorylation sites. OV350 was shown to acutely treat benzodiazepine-resistant status epilepticus (SE) in a kainite mouse model, providing preclinical proof-of-concept for this intravenous (IV) compound for treating refractory SE (RSE) and perhaps other medical emergencies, such as neonatal hypoxic-ischemic encephalopathy [HIE; where CLP290 has shown efficacy (Sullivan et al., 2021)] and acute psychosis (for which OV350 has shown efficacy in the phencyclidine model; recently shared via Ovid’s public website).

Another group of KCC2 target biology experts at Vanderbilt, who had previously contributed to the important discovery of potent and selective KCC2 inhibitors (Delpire et al., 2009; Delpire et al., 2012; Delpire and Weaver, 2016), also recently identified small molecule KCC2 potentiators. The Weaver/Delpire group Cl^–^ efflux screen (SuperClomeleon sensor) in HEK-293 cells with an inducible level of KCC2 expression identified selective KCC2 potentiator VU0500469, which also attenuated seizure-like activity in neuronal-glial co-cultures (Prael et al., 2022).

In addition to direct activators/potentiators, several labs have identified indirect KCC2 modulators that increase KCC2 function/expression. Liabeuf et al. (2017) screened molecules in Tl^+^ influx assay in HEK-KCC2 cells from the Prestwick Chemical Library consisting of off-patent approved drugs to identify a new KCC2 enhancer, prochlorperazine (blocks D2 dopamine receptors as well as other histaminergic, cholinergic, and noradrenergic receptors) which increased KCC2 activity, hyperpolarizes the ECl in motor neurons and alleviated spasticity in a rat spinal cord injury (SCI) model (Liabeuf et al., 2017), providing a potential drug-repurposing strategy. Tang et al. (2019) screened > 900 small molecules for their ability to increase KCC2 expression in KCC2 reporter human neurons, and found several hits including Flt3 inhibitor KW-2449 and GSK3β inhibitor BIO which treated deficits in Rett syndrome human neurons and/or animal models (Tang et al., 2019). Similarly, Wolfgang Liedtke’s group screened over 1,000 cancer drugs for KCC2 gene expression-enhancers in primary cortical neurons to identify another hit GSK3β inhibitor Kenpaullone (Yeo et al., 2021). Kenpaullone increases KCC2 expression, re-normalizes ECl (E_*GABA*_) in neurons, and has analgesic efficacy in neuropathic and bone cancer pain models (Yeo et al., 2021), as well as a sensory itch/dermatitis model (Yeo et al., 2022). Studies have demonstrated that TCB-2, an activator of 5-HT2A, increases KCC2 expression to treat neuropathic pain in the SCI model and mitigates the effects of stress on dysregulation of KCC2-dependent GABAergic signaling (Sánchez-Brualla et al., 2018; Kimmey et al., 2019). Other indirect expression enhancers of KCC2, including TrkB (e.g., Ana12) and WNK (e.g., WNK-463) inhibitors, have been shown to protect against drug-resistant severe seizures (Kang et al., 2015; Lee et al., 2022; Vyas et al., 2024), again demonstrating the utility of this KCC2 expression enhancer therapeutic approach. Finally, viral-mediated increases in KCC2 expression have been used to validate the therapeutic effects of small molecule treatments in animal models [e.g., AAV-KCC2 and CLP290 in SCI (Chen et al., 2018)], but also demonstrate the robustness and increasing therapeutic effects of chronic KCC2 upmodulation in epilepsy, pain, and SCI models (Li et al., 2016; Chen et al., 2018; Magloire et al., 2019), as well as providing proof-of-concept for the therapeutic utility of one-and-done viral KCC2 overexpression gene therapies.

## Clinical considerations for the development of KCC2-targeting therapeutics

KCC2 is a genetically validated target in human epilepsy and neurodevelopmental disorders (Fukuda and Watanabe, 2019), providing important human data to inform indication selection for first-in-class KCC2 therapeutics. Indeed, autosomal recessive inheritance of compound heterozygous pathogenic loss-of-function variants in the solute carrier family 12-member 5 (SLC12A5) gene is associated with the development of idiopathic generalized epilepsy, focal refractory epilepsy, and early infantile epileptic encephalopathy, with these patients also being diagnosed with intellectual disability, ASD as well as pharmacoresistance (Kahle et al., 2014b; Stödberg et al., 2015; Saitsu et al., 2016; Duy et al., 2019; Fukuda and Watanabe, 2019; Jarvela et al., 2024). Since SLC12A5 has been included in epilepsy genetic screening panels, the number of KCC2 mutant variants identified has exponentially grown in recent years (see ∼900 SLC12A5 variants identified to date on ClinVar). Previously, studies of loss-of-function KCC2 mutations in mice had also demonstrated the critical role of KCC2 in reducing seizure susceptibility (Woo et al., 2002; Tornberg et al., 2005; Silayeva et al., 2015; Pisella et al., 2019). Moreover, several studies using resected tissue from refractory epilepsy patients have demonstrated KCC2 hypofunction in these drug-resistant seizure-generating foci (Cohen et al., 2002; Palma et al., 2006; Huberfeld et al., 2007; Munakata et al., 2007; Muñoz et al., 2007; Eichler et al., 2008; Pallud et al., 2014; Donneger et al., 2023; Bakouh et al., 2024; Sosunov et al., 2024). Interestingly, a recent report found that two KCC2 potentiators, CLP257 and PCPZ, suppressed spontaneous epileptiform discharges in 10 out of 13 surgically-resected tissues from adult refractory focal epilepsy patients (Donneger et al., 2023), building on a previous study demonstrating that depolarizing GABAergic neurotransmission contributes to seizure propagation in these drug-resistant seizure foci of patients (Cohen et al., 2002). Therefore, the development of KCC2 potentiators to treat genetic and acquired epilepsies is robustly supported by genetic, clinical, and preclinical data.

Beyond epilepsies where genetic screening and therapeutic resection surgeries are more common, human genetic validation and patient tissue studies for non-epileptic neurological disorders involving acquired KCC2 hypofunction can be more difficult. As a result, despite a wide range of supportive preclinical evidence for KCC2 hypofunction in neurological disorders that has been reviewed in depth by others (Kaila et al., 2014; Duy et al., 2020; Tang, 2020; Belperio et al., 2022; Hegarty and Stanicka, 2022; Liedtke, 2022; Lam et al., 2023; Mcmoneagle et al., 2023; Pressey et al., 2023; Tomita et al., 2023; Mcardle et al., 2024) and outlined above, indication selection and prioritization can be more difficult to justify in these cases. The utilization of human neuron models derived from patient induced pluripotent cell lines (iPSCs) has allowed the demonstration of KCC2 hypofunction downstream of the monogenic causes of NDDs, such as *MECP2* mutant Rett syndrome neurons (Tang et al., 2016; Tang et al., 2019). Because KCC2 plays a critical role in brain maturation, with its expression exponentially increasing postnatally to establish inhibitory neurotransmission in the CNS, it is commonly implicated in the downstream E/I imbalance pathologies of NDDs, including Syngap1 haploinsufficiency disorder, Down syndrome, fragile X, tuberous sclerosis complex and focal cortical dysplasia (Talos et al., 2012; He et al., 2014; Kaila et al., 2014; Deidda et al., 2015; Tang et al., 2019; Verma et al., 2022; Bakouh et al., 2024; Sosunov et al., 2024). While these human iPSC models are particularly useful for examining the contribution of KCC2 hypofunction to particular NDDs/DEEs, because KCC2 is a mature neuron synaptic protein, it is important that the iPSC-derived neuronal differentiation protocols chosen for such studies enable sufficient KCC2 expression. In cases of adult-onset acquired neuropathologies, multiple preclinical reports over the last two decades have characterized the mechanisms by which KCC2 hypofunction in the dorsal sensory horn of the spinal cord leads to spinal disinhibition underlying chronic neuropathic pain (Coull et al., 2003; Prescott et al., 2006; Jolivalt et al., 2008; Kahle et al., 2014a; Li et al., 2019; Mapplebeck et al., 2019; Yeo et al., 2021; Aby et al., 2022; Liedtke, 2022). In this case because of KCC2 hypofunction, impaired GABAergic inhibition along ascending sensory/nociceptive and descending modulatory pathways (e.g., brain stem-derived and corticospinal pathways) facilitates chronic pain in a pathological process known as CNS sensitization. The intellectual disability and ASD/NDD comorbidities of biallelic KCC2 mutant patients preclude assessment of altered pain processing in these individuals, but KCC2 hypofunction in the dorsal spinal cord was demonstrated in a human neuropathic pain model (Dedek et al., 2019).

In the absence of human genetic, tissue, or model validations, clinical biomarkers of KCC2 hypofunction pathology can also be used to facilitate bridging the preclinical-to-clinical translational gap. In this regard, the rate-dependent depression of the H-reflex (HRDD) is a biomarker of GABAergic disinhibition within the spinal cord. It also serves as a pharmacodynamic biomarker of KCC2 function in spinal neurons: (1) HRDD is impaired by reduced KCC2 expression or KCC2 inhibition; and (2) impaired HRDD can be restored by KCC2 potentiation treatment in animal models of chronic pain, SCI, and spasticity (Jolivalt et al., 2008; Boulenguez et al., 2010; Côté et al., 2014; Lee-Kubli and Calcutt, 2014; Toda et al., 2014; Liabeuf et al., 2017; Marshall et al., 2017; Lee-Kubli et al., 2018; Tom et al., 2018; Bilchak et al., 2021; Lee-Kubli et al., 2021; Bilchak et al., 2023; Malloy and Côté, 2024; Pan et al., 2024). Interestingly, 30–40% of patients with diabetic neuropathic pain have impaired HRDD, reflecting spinal disinhibition-driven chronic pain, which directly correlates with pain phenotypes and can predict analgesic efficacy in patients (Millán-Guerrero et al., 2012; Worthington et al., 2021b; Worthington et al., 2021a; Zhou et al., 2022; Marshall et al., 2023). Therefore, HRDD is an objective electrophysiological biomarker of KCC2 hypofunction, impaired GABAergic inhibition, and neuropathic pain, and may serve as a pharmacodynamic and disease-relevant biomarker of KCC2 potentiation therapy in patients. Other translational biomarkers relevant for measuring KCC2 function in the CNS include other electrophysiological measures, such as qEEG, which have been profiled for other CNS agents (Groeneveld and Hay, 2016) and may enable human dose determination in the clinic for novel KCC2 potentiator compounds. Especially event-related potentials (ERPs) that measure impaired inhibition in CNS circuits in healthy volunteers or patients (Premoli et al., 2014; Cecchi et al., 2023). It will be interesting to compare and contrast the clinical qEEG biomarker profile and ERPs of KCC2 potentiators to other ASMs and analgesics, particularly GABA_*A*_R PAMs. Finally, there have been some reports of KCC2 biomarkers in the CSF of patients (Duarte et al., 2013), which may be particularly relevant for KCC2-expression enhancer treatments.

For a first-in-human efficacy study, the most direct method for identifying patients with KCC2 hypofunction would be genetic testing for pathogenic KCC2 variants, which are typically identified when individuals recessively inherit two compound heterozygous loss-of-function mutants. While this approach is logical, it would be important to ensure that the mechanism of action of the KCC2 potentiator treatment could rescue the function of these mutant proteins. For example, agents that restore surface expression of KCC2 would likely not rescue the function of KCC2 variants with loss-of-function mutations in the transmembrane chloride transport core (Fukuda and Watanabe, 2019). However, since the mutational landscape is being characterized, and a growing number of SLC12A5 variants are being identified, groups are beginning to understand the pathogenic consequences of heterozygous KCC2 mutations, which have been associated with psychiatric (e.g., schizophrenia) and mood disorders in GWAS studies recently (Hyde et al., 2011; Tao et al., 2012; Merner et al., 2015). In these cases, potentiating the function of the intact allele of KCC2 could confer a targeted therapeutic benefit, irrespective of the ability of the KCC2 therapeutic to correct the function of the mutant allele.

## Discussion

Despite progress in the number of approved medicines for epilepsies, psychiatry, and other excitation/inhibition (E/I) imbalance CNS disorders in recent decades, a significant proportion of patients remain drug-resistant or cannot tolerate the disabling side effects of these first-line medications. Current marketed drugs typically have overlapping mechanisms that target ion channels or neurotransmitter systems to globally suppress brain activity. Alternatively, by fine-tuning ionic homeostasis and physiological inhibition within the CNS, KCC2-potentiating therapeutics may have superior tolerability and can address this urgent, unmet need by converting patients from drug-resistant to drug-sensitive. To realize the full therapeutic potential of KCC2, we need to build a portfolio of medicines that utilize novel and differentiated mechanisms of action, which also match the modality to patient needs, to restore physiological inhibition to the CNS to provide meaningful and targeted clinical benefits to patients.

Tremendous progress has been made toward the development of first-in-class therapeutics that target KCC2 in recent years. Three broad classes of KCC2 therapeutics have emerged to date: (1) Small molecule direct KCC2 activators/potentiators (e.g., AXN-027; CLPs; OV350; VU0500469); (2) Small molecule indirect KCC2 expression enhancers (e.g., PCPZ and repurposed kinase inhibitors); and (3) Viral-mediated KCC2 overexpression gene therapies (e.g., Lent-viral KCC2 in development by NxGen Medicine for chronic lower back pain). The CNS neuron-specific expression profile of KCC2 makes it a very attractive target for a CNS-penetrant small molecule, precluding the need to choose the gene therapy approach as long as chronic treatment with the oral KCC2 potentiator small molecule is feasible, well-tolerated and can persistently correct the KCC2 hypofunction pathology within the CNS. Moreover, the target biology of KCC2 should avoid the cognitive/behavioral side effects of existing approved drug mechanisms that directly hyperpolarize neuronal RMPs. By priming CNS neurons to be hyperpolarized by GABA and glycine at inhibitory synapses, KCC2 potentiation therapy should enable physiological levels of CNS circuit inhibition, without sedation/somnolence. In regards to risks of chronic oral KCC2 potentiation therapy, no human pathogenic gain-of-function KCC2 mutations have been identified to date, while no clinical signs have been reported in rodent studies of chronic KCC2 potentiation therapy or long-term viral/CRISPR mediated KCC2 overexpression (Li et al., 2016; Chen et al., 2018; Magloire et al., 2019; Cheung et al., 2022). Importantly, KCC2 gain-of-function mutant mice (T906A/T1007A) exhibit normal brain morphology and neuronal network excitability, with improved memory function and increased sociability and were protected from seizures (Moore et al., 2018; Moore et al., 2019). In fact, chronic KCC2 potentiation therapy has been shown to increase efficacy over time (Li et al., 2016; Chen et al., 2018), as opposed to the tolerance/tachyphylaxis observed for repeated treatment with GABA_*A*_R agonists/PAMs (Woldman et al., 2019), which may be due to KCC2 role in synaptic spine density and strengthening of inhibitory neuronal circuits (Li et al., 2007; Seja et al., 2012; Fiumelli et al., 2013; Kaila et al., 2014; Puskarjov et al., 2014; Awad et al., 2018). Interestingly, in addition to the potential of KCC2 in treating neurodegenerative diseases, KCC2 hypofunction has also been implicated in age-related memory decline (Ferando et al., 2016; Keramidis et al., 2023; Khademullah et al., 2023). Thus, the potential safety, tolerability and precise efficacy of KCC2 potentiation therapy is ideally suited for rapid titration and oral chronic treatment, as required and preferred for many epilepsy, neuropathic pain and other patients with chronic or age-related neurological disorders.

In conclusion, we are close to realizing the first ion homeostatic medicine for the CNS that enables GABA signaling without global inhibition and debilitating sedation, with two development candidates for KCC2-targeting therapeutics nearing the clinic: (1) Axonis Therapeutics first-in-class oral KCC2 potentiator AXN-027 is set to begin first-in-human clinical trials in healthy volunteers in Q4 2024, and (2) Ovid Therapeutics plan to test their first-in-class IV KCC2 activator OV350 in H1 2025. Placebo-controlled proof-of-concept clinical trials in (1) acute psychosis for IV OV350, and (2) chronic epilepsy and pain trials for oral AXN-027 will also pave the way for the next generation of KCC2 potentiation therapies, such as KCC2 expression-enhancing approaches, KCC2 gene therapies or alternative formulations/routes of administration tailored to patient’s needs (e.g., oral solution in ASDs/NDDs/DEEs; nasal spray for acute pain/seizures). To improve novel drug discovery, next generation screening assays which precisely measure the direct activity of KCC2, but not other chloride transporter/flux mechanism, in mature neurons at scale are needed. Moreover, the development of additional clinical biomarkers of KCC2 hypofunction and pharmacodynamic biomarkers of KCC2 potentiation therapy will enable further indication selection and patient stratification to realize the full potential of these therapeutics to address CNS disinhibition and drug-resistance in neurological disorder patients with unmet needs, likely providing a more tolerable and precise medication to address their chronic pathologies. Within the next decade, targeted KCC2 therapeutics are poised to transform precision medicine for neurological disorders.

## Data Availability

The original contributions presented in this study are included in this article/supplementary material, further inquiries can be directed to the corresponding author.

## References

[B1] Aby F. Lorenzo L. E. Grivet Z. Bouali-Benazzouz R. Martin H. Valerio S. (2022). Switch of serotonergic descending inhibition into facilitation by a spinal chloride imbalance in neuropathic pain. *Sci. Adv.* 8:eabo0689. 10.1126/sciadv.abo0689 35895817 PMC9328683

[B2] Awad P. N. Amegandjin C. A. Szczurkowska J. Carriço J. N. Fernandes do Nascimento A. S. Baho E. (2018). KCC2 regulates dendritic spine formation in a brain-region specific and BDNF dependent manner. *Cereb. Cortex* 28 4049–4062. 10.1093/cercor/bhy198 30169756 PMC6188549

[B3] Bakouh N. Castaño-Martín R. Metais A. Dan E. L. Balducci E. Chhuon C. (2024). Chloride deregulation and GABA depolarization in MTOR related malformations of cortical development. *Brain* awae262. [Online ahead of print]. 10.1093/brain/awae262 39106285 PMC11788215

[B4] Belperio G. Corso C. Duarte C. B. Mele M. (2022). Molecular mechanisms of epilepsy: the role of the chloride transporter KCC2. *J. Mol. Neurosci.* 72 1500–1515. 10.1007/s12031-022-02041-7 35819636

[B5] Bilchak J. N. Caron G. Danner S. M. Côté M.-P. (2023). KCC2 enhancers normalize reflex responses and improve locomotor function after chronic spinal cord injury. *bioRxiv [Preprint]* 10.1101/2023.10.21.563363 PMC12487610 40906931

[B6] Bilchak J. N. Yeakle K. Caron G. Malloy D. Côté M. P. (2021). Enhancing KCC2 activity decreases hyperreflexia and spasticity after chronic spinal cord injury. *Exp. Neurol.* 338:113605. 10.1016/j.expneurol.2021.113605 33453210 PMC7904648

[B7] Boulenguez P. Liabeuf S. Bos R. Bras H. Jean-Xavier C. Brocard C. (2010). Down-regulation of the potassium-chloride cotransporter KCC2 contributes to spasticity after spinal cord injury. *Nat. Med.* 16 302–307. 20190766 10.1038/nm.2107

[B8] Cecchi M. Adachi M. Basile A. Buhl D. L. Chadchankar H. Christensen S. (2023). Validation of a suite of ERP and QEEG biomarkers in a pre-competitive, industry-led study in subjects with schizophrenia and healthy volunteers. *Schizophr. Res.* 254 178–189. 10.1016/j.schres.2023.02.018 36921403

[B9] Chen B. Li Y. Yu B. Zhang Z. Brommer B. Williams P. R. (2018). Reactivation of dormant relay pathways in injured spinal cord by KCC2 manipulations. *Cell* 521–535. 10.1016/j.cell.2018.06.005 30033363 PMC6063786

[B10] Cheung D. L. Cooke M. J. Goulton C. S. Chaichim C. Cheung L. F. Khoshaba A. (2022). Global transgenic upregulation of KCC2 confers enhanced diazepam efficacy in treating sustained seizures. *Epilepsia* 63 e15–e22. 10.1111/epi.17097 34791657

[B11] Cohen I. Navarro V. Clemenceau S. Baulac M. Miles R. (2002). On the origin of interictal activity in human temporal lobe epilepsy *in vitro*. *Science* 298 1418–1421. 12434059 10.1126/science.1076510

[B12] Côté M.-P. Gandhi S. Zambrotta M. Houlé J. D. (2014). Exercise modulates chloride homeostasis after spinal cord injury. *J. Neurosci.* 34 8976–8987. 24990918 10.1523/JNEUROSCI.0678-14.2014 PMC6608257

[B13] Coull J. A. Boudreau D. Bachand K. Prescott S. A. Nault F. Sík A. (2003). Trans-synaptic shift in anion gradient in spinal lamina I neurons as a mechanism of neuropathic pain. *Nature* 424 938–942. 10.1038/nature01868 12931188

[B14] Dedek A. Xu J. Kandegedara C. M. Lorenzo L. É Godin A. G. De Koninck Y. (2019). Loss of STEP61 couples disinhibition to N-methyl-d-aspartate receptor potentiation in rodent and human spinal pain processing. *Brain* 142 1535–1546. 10.1093/brain/awz105 31135041 PMC6536915

[B15] Deidda G. Parrini M. Naskar S. Bozarth I. F. Contestabile A. Cancedda L. (2015). Reversing excitatory GABAAR signaling restores synaptic plasticity and memory in a mouse model of Down syndrome. *Nat. Med.* 21 318–326. 10.1038/nm.3827 25774849

[B16] Delpire E. Weaver C. D. (2016). Challenges of finding novel drugs targeting the K-Cl cotransporter. *ACS Chem. Neurosci.* 7 1624–1627. 10.1021/acschemneuro.6b00366 27998063 PMC5473358

[B17] Delpire E. Baranczak A. Waterson A. G. Kim K. Kett N. Morrison R. D. (2012). Further optimization of the K-Cl cotransporter KCC2 antagonist ML077: development of a highly selective and more potent in vitro probe. *Bioorganic Med. Chem. Lett.* 22 4532–4535. 10.1016/j.bmcl.2012.05.126 22727639 PMC3389279

[B18] Delpire E. Days E. Lewis L. Mi D. Kim K. Lindsley C. (2009). Small-molecule screen identifies inhibitors of the neuronal K-Cl cotransporter KCC2. *Proc. Natl. Acad. Sci. U.S.A.* 106 5383–5388. 10.1073/pnas.0812756106 19279215 PMC2654392

[B19] Donneger A. F. Zanin A. Besson J. Kadiri Y. Pagan C. David N. (2023). Enhancing KCC2 function reduces interictal activity and prevents seizures in mesial temporal lobe epilepsy. *bioRxiv [Preprint]* 10.1101/2023.09.16.557753 PMC12974483 41774803

[B20] Duarte S. T. Armstrong J. Roche A. Ortez C. Pérez A. O’Callaghan Mdel M. (2013). Abnormal expression of cerebrospinal fluid cation chloride cotransporters in patients with rett syndrome. *PLoS One* 8:e68851. 10.1371/journal.pone.0068851 23894354 PMC3716803

[B21] Duy P. Q. David W. B. Kahle K. T. Chevy Q. (2019). Identification of KCC2 mutations in human epilepsy suggests strategies for therapeutic transporter modulation. *Front. Cell. Neurosci.* 13:515. 10.3389/fncel.2019.00515 31803025 PMC6873151

[B22] Duy P. Q. He M. He Z. Kahle K. T. (2020). Preclinical insights into therapeutic targeting of KCC2 for disorders of neuronal hyperexcitability. *Expert Opin. Ther. Targets* 24 629–637. 10.1080/14728222.2020.1762174 32336175 PMC8104019

[B23] Dzhala V. I. Staley K. J. (2021). KCC2 chloride transport contributes to the termination of ictal epileptiform activity. *eNeuro* 8 1–16. 10.1523/ENEURO.0208-20.2020 33239270 PMC7986536

[B24] Eichler S. A. Kirischuk S. Jüttner R. Schaefermeier P. K. Legendre P. Lehmann T. N. (2008). Glycinergic tonic inhibition of hippocampal neurons with depolarizing GABAergic transmission elicits histopathological signs of temporal lobe epilepsy. *J. Cell. Mol. Med.* 12 2848–2866. 10.1111/j.1582-4934.2008.00357.x 19210758 PMC3828897

[B25] Ferando I. Faas G. C. Mody I. (2016). Diminished KCC2 confounds synapse specificity of LTP during senescence. *Nat. Neurosci.* 19 1197–1200. 10.1038/nn.4357 27500406 PMC5003660

[B26] Ferrini F. Lorenzo L.-E. Godin A. G. Le Quang M. De Knoninck Y. (2017). Enhancing KCC2 function counteracts morphine-induced hyperalgesia. *Sci. Rep.* 7:3870. 10.1038/s41598-017-04209-3 28634406 PMC5478677

[B27] Fiumelli H. Briner A. Puskarjov M. Blaesse P. Belem B. J. Dayer A. G. (2013). An ion transport-independent role for the cation-chloride cotransporter kcc2 in dendritic spinogenesis *in vivo*. *Cereb. Cortex* 23 378–388. 10.1093/cercor/bhs027 22345354

[B28] Fukuda A. Watanabe M. (2019). Pathogenic potential of human SLC12A5 variants causing KCC2 dysfunction. *Brain Res.* 1710 1–7. 30576625 10.1016/j.brainres.2018.12.025

[B29] Gagnon M. Bergeron M. J. Lavertu G. Castonguay A. Tripathy S. Bonin R. P. (2013). Chloride extrusion enhancers as novel therapeutics for neurological diseases. *Nat. Med.* 19 1524–1528. 24097188 10.1038/nm.3356 PMC4005788

[B30] Groeneveld G. J. Hay J. L. (2016). Drug discovery and the BBB Measuring blood – brain barrier penetration using the NeuroCart, a CNS test battery. *Drug Discov. Today Technol.* 20 27–34. 10.1016/j.ddtec.2016.07.004 27986220

[B31] He Q. Nomura T. Xu J. Contractor A. (2014). The developmental switch in GABA polarity is delayed in fragile X mice. *J. Neurosci.* 34 446–450. 10.1523/JNEUROSCI.4447-13.2014 24403144 PMC6608154

[B32] Hegarty S. V. Stanicka J. (2022). K + /Cl - co-transporter-2 upmodulation: a multi-modal therapy to treat spinal cord injury. *Neural Regen. Res.* 17:1984. 10.4103/1673-5374.335155 35142685 PMC8848609

[B33] Hinz L. Torrella Barrufet J. Heine V. M. (2019). KCC2 expression levels are reduced in post mortem brain tissue of Rett syndrome patients. *Acta Neuropathol. Commun.* 7:196. 10.1186/s40478-019-0852-x 31796123 PMC6892240

[B34] Huberfeld G. Wittner L. Clemenceau S. Baulac M. Kaila K. Miles R. (2007). Perturbed chloride homeostasis and GABAergic signaling in human temporal lobe epilepsy. *J. Neurosci.* 27 9866–9873. 10.1523/JNEUROSCI.2761-07.2007 17855601 PMC6672644

[B35] Hyde T. M. Lipska B. K. Ali T. Mathew S. V. Law A. J. Metitiri O. E. (2011). Expression of GABA signaling molecules KCC2, NKCC1, and GAD1 in cortical development and schizophrenia. *J. Neurosci.* 31 11088–11095. 10.1523/JNEUROSCI.1234-11.2011 21795557 PMC3758549

[B36] Jarvela V. Hamze M. Komulainen-Ebrahim J. Rahikkala E. Piispala J. Kallio M. (2024). A novel pathogenic SLC12A5 missense variant in epilepsy of infancy with migrating focal seizures causes impaired KCC2 chloride extrusion. *Front. Mol. Neurosci.* 17:1372662. 10.3389/fnmol.2024.1372662 38660387 PMC11039960

[B37] Jarvis R. Josephine Ng S. F. Nathanson A. J. Cardarelli R. A. Abiraman K. Wade F. (2023). Direct activation of KCC2 arrests benzodiazepine refractory status epilepticus and limits the subsequent neuronal injury in mice. *Cell Rep. Med.* 4:100957. 10.1016/j.xcrm.2023.100957 36889319 PMC10040380

[B38] Jolivalt C. G. Lee C. A. Ramos K. M. Calcutt N. A. (2008). Allodynia and hyperalgesia in diabetic rats are mediated by GABA and depletion of spinal potassium-chloride co-transporters. *Pain* 140 48–57. 10.1016/j.pain.2008.07.005 18755547 PMC2593464

[B39] Kahle K. T. Khanna A. Clapham D. E. Woolf C. J. (2014a). Therapeutic restoration of spinal inhibition via druggable enhancement of potassium-chloride cotransporter KCC2–mediated chloride extrusion in peripheral neuropathic pain. *JAMA Neurol.* 71:640. 10.1001/jamaneurol.2014.21 24615367 PMC4465580

[B40] Kahle K. T. Merner N. D. Friedel P. Silayeva L. Liang B. Khanna A. (2014b). Genetically encoded impairment of neuronal KCC 2 cotransporter function in human idiopathic generalized epilepsy. *EMBO Rep.* 15 766–774. 10.15252/embr.201438840 24928908 PMC4196980

[B41] Kaila K. Price T. J. Payne J. A. Puskarjov M. Voipio J. (2014). Cation-chloride cotransporters in neuronal development, plasticity and disease. *Nat. Rev. Neurosci.* 15 637–654. 25234263 10.1038/nrn3819 PMC4294553

[B42] Kang S. K. Johnston M. V. Kadam S. D. (2015). Acute TrkB inhibition rescues phenobarbital-resistant seizures in a mouse model of neonatal ischemia. *Eur. J. Neurosci.* 42 2792–2804. 10.1111/ejn.13094 26452067 PMC4715496

[B43] Keramidis I. McAllister B. B. Bourbonnais J. Wang F. Isabel D. Rezaei E. (2023). Restoring neuronal chloride extrusion reverses cognitive decline linked to Alzheimer’s disease mutations. *Brain* 146 4903–4915. 10.1093/brain/awad250 37551444 PMC10690023

[B44] Khademullah C. S. Bourbonnais J. Chaineau M. M. Castellanos-Montiel M. J. Keramidis I. Legault A. (2023). KCC2 as a novel biomarker and therapeutic target for motoneuron degenerative disease. *bioRxiv [Preprint]* 10.1101/2023.08.24.554410

[B45] Kimmey B. A. Ostroumov A. Dani J. A. (2019). 5-HT2A receptor activation normalizes stress-induced dysregulation of GABAergic signaling in the ventral tegmental area. *Proc. Natl. Acad. Sci. U.S.A.* 116 27028–27034. 10.1073/pnas.1911446116 31806759 PMC6936697

[B46] Lam P. Newland J. Faull R. L. M. (2023). Cation-chloride cotransporters KCC2 and NKCC1 as therapeutic targets in neurological and neuropsychiatric disorders. *Molecules* 28:2440. 10.3390/molecules28031344 PMC9920462 36771011

[B47] Lee H. H. C. Deeb T. Z. Walker J. A. Davies P. A. Moss S. J. (2011). NMDA receptor activity downregulates KCC2 resulting in depolarizing GABAA receptor-mediated currents. *Nat. Neurosci.* 14 736–743. 10.1038/nn.2806 21532577 PMC3102766

[B48] Lee K. L. Abiraman K. Lucaj C. Ollerhead T. Brandon N. Deeb T. (2022). Inhibiting with-no-lysine kinases enhances K + / Cl – cotransporter 2 activity and limits status epilepticus. *Brain* 145 950–963. 10.1093/brain/awab343 34528073 PMC9050525

[B49] Lee-Kubli C. A. G. Calcutt N. A. (2014). Altered rate-dependent depression of the spinal H-reflex as an indicator of spinal disinhibition in models of neuropathic pain. *Pain* 155 250–260. 10.1016/j.pain.2013.10.001 24103402 PMC3946970

[B50] Lee-Kubli C. A. Zhou X. Jolivalt C. G. Calcutt N. A. (2021). Pharmacological modulation of rate-dependent depression of the spinal h-reflex predicts therapeutic efficacy against painful diabetic neuropathy. *Diagnostics* 11:283. 10.3390/diagnostics11020283 33670344 PMC7917809

[B51] Lee-Kubli C. Marshall A. G. Malik R. A. Calcutt N. A. (2018). The H-Reflex as a biomarker for spinal disinhibition in painful diabetic neuropathy. *Curr. Diab. Rep.* 18:283. 10.1007/s11892-018-0969-5 29362940 PMC6876556

[B52] Li C. Lei Y. Tian Y. Xu S. Shen X. Wu H. (2019). The etiological contribution of GABAergic plasticity to the pathogenesis of neuropathic pain. *Mol. Pain* 15:1744806919847366. 10.1177/1744806919847366 30977423 PMC6509976

[B53] Li H. Khirug S. Cai C. Ludwig A. Blaesse P. Kolikova J. (2007). KCC2 interacts with the dendritic cytoskeleton to promote spine development. *Neuron* 56 1019–1033. 18093524 10.1016/j.neuron.2007.10.039

[B54] Li J. Zhang L. Xu C. Lin Y. H. Zhang Y. Wu H. Y. (2020). Prolonged use of NMDAR antagonist develops analgesic tolerance in neuropathic pain via nitric oxide reduction-induced GABAergic disinhibition. *Neurotherapeutics* 17 1016–1030. 10.1007/s13311-020-00883-w 32632774 PMC7609518

[B55] Li L. Chen S. R. Chen H. Wen L. Hittelman W. N. Xie J. D. (2016). Chloride homeostasis critically regulates synaptic NMDA receptor activity in neuropathic pain. *Cell Rep.* 15 1376–1383. 27160909 10.1016/j.celrep.2016.04.039 PMC4871741

[B56] Liabeuf S. Stuhl-Gourmand L. Gackière F. Mancuso R. Sanchez Brualla I. Marino P. (2017). Prochlorperazine increases KCC2 function and reduces spasticity after spinal cord injury. *J. Neurotrauma* 34 3397–3406. 28747093 10.1089/neu.2017.5152

[B57] Liedtke W. (2022). Long March toward safe and effective analgesia by enhancing gene expression of Kcc2: first steps taken. *Front. Mol. Neurosci.* 15:865600. 10.3389/fnmol.2022.865600 35645734 PMC9137411

[B58] Lorenzo L. E. Godin A. Ferrini F. Bachand K. Plasencia-Fernandez I. Labrecque S. (2020). Enhancing neuronal chloride extrusion rescues α2/α3 GABAA-mediated analgesia in neuropathic pain. *Nat. Commun.* 11:869. 10.1038/s41467-019-14154-6 PMC7018745 32054836

[B59] Magloire V. Cornford J. Lieb A. Kullmann D. M. Pavlov I. (2019). KCC2 overexpression prevents the paradoxical seizure-promoting action of somatic inhibition. *Nat. Commun.* 10:1225. 10.1038/s41467-019-08933-4 30874549 PMC6420604

[B60] Malloy D. C. Côté M.-P. (2024). Multi-session transcutaneous spinal cord stimulation prevents chloride homeostasis imbalance and the development of hyperreflexia after spinal cord injury in rat. *Exp. Neurol.* 376:114754. 10.1016/j.expneurol.2024.114754 38493983 PMC11519955

[B61] Mapplebeck J. C. S. Lorenzo L. E. Lee K. Y. Gauthier C. Muley M. M. De Koninck Y. (2019). Chloride dysregulation through downregulation of KCC2 mediates neuropathic pain in both sexes. *Cell Rep.* 28 590–596.e4. 10.1016/j.celrep.2019.06.059 31315039

[B62] Marshall A. G. Lee-Kubli C. Azmi S. Zhang M. Ferdousi M. Mixcoatl-Zecuatl T. (2017). Spinal disinhibition in experimental and clinical painful diabetic neuropathy. *Diabetes* 66 1380–1390. 28202580 10.2337/db16-1181 PMC5399611

[B63] Marshall A. Kalteniece A. Ferdousi M. Azmi S. Jude E. B. Adamson C. (2023). Spinal disinhibition: evidence for a hyperpathia phenotype in painful diabetic neuropathy. *Brain Commun.* 5:fcad051. 10.1093/braincomms/fcad051 36938521 PMC10016414

[B64] Mcardle C. J. Arnone A. A. Heaney C. F. Raab-graham K. F. Raab-graham K. F. (2024). A paradoxical switch: the implications of excitatory GABAergic signaling in neurological disorders. *Front. Psychiatry* 14:1296527. 10.3389/fpsyt.2023.1296527 38268565 PMC10805837

[B65] Mcmoneagle E. Zhou J. Zhang S. Huang W. Josiah S. S. Ding K. (2023). Neuronal K -Cl - cotransporter KCC2 as a promising drug target for epilepsy treatment. *Acta Pharmacol. Sin.* 45 1–22. 10.1038/s41401-023-01149-9 37704745 PMC10770335

[B66] Merner N. D. Chandler M. Bourassa C. Liang B. Khanna A. Dion P. (2015). Regulatory domain or CPG site variation in SLC12A5, encoding the chloride transporter KCC2, in human autism and schizophrenia. *Front. Cell. Neurosci.* 9:386. 10.3389/fncel.2015.00386 26528127 PMC4600830

[B67] Millán-Guerrero R. Trujillo-Hernández B. Isais-Millán S. Prieto-Díaz-Chávez E. Vásquez C. Caballero-Hoyos J. (2012). H-reflex and clinical examination in the diagnosis of diabetic polyneuropathy. *J. Int. Med. Res.* 40 694–700. 22613432 10.1177/147323001204000233

[B68] Moore Y. E. Conway L. C. Wobst H. J. Brandon N. J. Deeb T. Z. Moss S. J. (2019). Developmental regulation of KCC2 phosphorylation has long-term impacts on cognitive function. *Front. Mol. Neurosci.* 12:173. 10.3389/fnmol.2019.00173 31396048 PMC6664008

[B69] Moore Y. E. Deeb T. Z. Chadchankar H. Brandon N. J. Moss S. J. (2018). Potentiating KCC2 activity is sufficient to limit the onset and severity of seizures. *Proc. Natl. Acad. Sci. U.S.A.* 115 10166–10171. 10.1073/pnas.1810134115 30224498 PMC6176565

[B70] Munakata M. Watanabe M. Otsuki T. Nakama H. Arima K. Itoh M. (2007). Altered distribution of KCC2 in cortical dysplasia in patients with intractable epilepsy. *Epilepsia* 48 837–844. 17284302 10.1111/j.1528-1167.2006.00954.x

[B71] Muñoz A. Méndez P. DeFelipe J. Alvarez-Leefmans F. J. (2007). Cation-chloride cotransporters and GABA-ergic innervation in the human epileptic hippocampus. *Epilepsia* 48 663–673. 10.1111/j.1528-1167.2007.00986.x 17319917

[B72] Paige C. Plasencia-Fernandez I. Kume M. Papalampropoulou-Tsiridou M. Lorenzo L. E. David E. T. (2022). A female-specific role for Calcitonin Gene-Related Peptide (CGRP) in rodent pain models. *J. Neurosci.* 42 1930–1944. 35058371 10.1523/JNEUROSCI.1137-21.2022 PMC8916765

[B73] Pallud J. Le Van Quyen M. Bielle F. Pellegrino C. Varlet P. Cresto N. (2014). Cortical GABAergic excitation contributes to epileptic activities around human glioma. *Sci. Transl. Med.* 6:244ra89. 10.1126/scitranslmed.3008065 25009229 PMC4409113

[B74] Palma E. Amici M. Sobrero F. Spinelli G. Di Angelantonio S. Ragozzino D. (2006). Anomalous levels of Cl- transporters in the hippocampal subiculum from temporal lobe epilepsy patients make GABA excitatory. *Proc. Natl. Acad. Sci. U.S.A.* 103 8465–8468. 10.1073/pnas.0602979103 16709666 PMC1482515

[B75] Pan Y. Talifu Z. Wang X. X. Ke H. Zhang C. J. Xu X. (2024). Combined use of CLP290 and bumetanide alleviates neuropathic pain and its mechanism after spinal cord injury in rats. *CNS Neurosci. Ther.* 30:e70045. 10.1111/cns.70045 39267289 PMC11393004

[B76] Pisella L. I. Gaiarsa J. L. Diabira D. Zhang J. Khalilov I. Duan J. (2019). Impaired regulation of KCC2 phosphorylation leads to neuronal network dysfunction and neurodevelopmental pathology. *Sci. Signal.* 12: eaay0300. 10.1126/scisignal.aay0300 31615899 PMC7192243

[B77] Prael F. J. Kim K. Du Y. Spitznagel B. D. Sulikowski G. A. Delpire E. (2022). Discovery of small molecule KCC2 potentiators which attenuate *in vitro* seizure-like activity in cultured neurons. *Front. Cell Dev. Biol.* 10:912812. 10.3389/fcell.2022.912812 35813195 PMC9263442

[B78] Premoli I. Castellanos N. Rivolta D. Belardinelli P. Bajo R. Zipser C. (2014). TMS-EEG signatures of GABAergic neurotransmission in the human cortex. *J. Neurosci.* 34 5603–5612. 24741050 10.1523/JNEUROSCI.5089-13.2014 PMC6608220

[B79] Prescott S. A. Sejnowski T. J. De Koninck Y. (2006). Reduction of anion reversal potential subverts the inhibitory control of firing rate in spinal lamina I neurons: towards a biophysical basis for neuropathic pain. *Mol. Pain* 2:32. 10.1186/1744-8069-2-32 17040565 PMC1624821

[B80] Pressey J. C. de Saint-Rome M. Raveendran V. A. Woodin M. A. (2023). Chloride transporters controlling neuronal excitability. *Physiol. Rev.* 103 1095–1135. 36302178 10.1152/physrev.00025.2021

[B81] Puskarjov M. Ahmad F. Kaila K. Blaesse P. (2012). Activity-dependent cleavage of the K-Cl cotransporter KCC2 mediated by calcium-activated protease calpain. *J. Neurosci.* 32 11356–11364. 10.1523/JNEUROSCI.6265-11.2012 22895718 PMC6621186

[B82] Puskarjov M. Seja P. Heron S. E. Williams T. C. Ahmad F. Iona X. (2014). A variant of KCC2 from patients with febrile seizures impairs neuronal Cl- extrusion and dendritic spine formation. *EMBO Rep.* 15 723–729. 10.1002/embr.201438749 24668262 PMC4197883

[B83] Richardson R. J. Petrou S. Bryson A. (2024). Established and emerging GABA A receptor pharmacotherapy for epilepsy. *Front. Pharmacol.* 15:1341472. 10.3389/fphar.2024.1341472 38449810 PMC10915249

[B84] Rivera C. Li H. Thomas-Crusells J. Lahtinen H. Viitanen T. Nanobashvili A. (2002). BDNF-induced TrkB activation down-regulates the K+-Cl- cotransporter KCC2 and impairs neuronal Cl- extrusion. *J. Cell Biol.* 159 747–752. 10.1083/jcb.200209011 12473684 PMC2173387

[B85] Saitsu H. Watanabe M. Akita T. Ohba C. Sugai K. (2016). Impaired neuronal KCC2 function by biallelic SLC12A5 mutations in migrating focal seizures and severe developmental delay. *Sci. Rep.* 6:30072. 10.1038/srep30072 27436767 PMC4951812

[B86] Sánchez-Brualla I. Boulenguez P. Brocard C. Liabeuf S. Viallat-Lieutaud A. Navarro X. (2018). Activation of 5-HT 2A receptors restores KCC2 function and reduces neuropathic pain after spinal cord injury. *Neuroscience* 387 48–57. 10.1016/j.neuroscience.2017.08.033 28844001

[B87] Seja P. Schonewille M. Spitzmaul G. Badura A. Klein I. Rudhard Y. (2012). Raising cytosolic Cl - in cerebellar granule cells affects their excitability and vestibulo-ocular learning. *EMBO J.* 31 1217–1230. 10.1038/emboj.2011.488 22252133 PMC3297995

[B88] Shi J. Xin H. Shao Y. Dai S. Tan N. Li Z. (2023). CRISPR-based KCC2 upregulation attenuates drug-resistant seizure in mouse models of epilepsy. *Ann. Neurol.* 94 91–105. 10.1002/ana.26656 37014252

[B89] Shimizu-Okabe C. Tanaka M. Matsuda K. Mihara T. Okabe A. Sato K. (2011). KCC2 was downregulated in small neurons localized in epileptogenic human focal cortical dysplasia. *Epilepsy Res.* 93 177–184. 10.1016/j.eplepsyres.2010.12.008 21256718

[B90] Silayeva L. Deeb T. Z. Hines R. M. Kelley M. R. Munoz M. B. Lee H. H. (2015). KCC2 activity is critical in limiting the onset and severity of status epilepticus. *Proc. Natl. Acad. Sci. U. S. A.* 112 3523–3528. 10.1073/pnas.1415126112 25733865 PMC4371976

[B91] Sosunov A. A. McKhann Ii G. Tang G. Goldman J. E. (2024). Cytoplasmic vacuolization and ectopic formation of perineuronal nets are characteristic pathologies of cytomegalic neurons in tuberous sclerosis. *J. Neuropathol. Exp. Neurol.* 83 1047–1059. 10.1093/jnen/nlae079 39024216 PMC11576550

[B92] Stödberg T. McTague A. Ruiz A. J. Hirata H. Zhen J. Long P. (2015). Mutations in SLC12A5 in epilepsy of infancy with migrating focal seizures. *Nat. Commun.* 6:8038. 10.1038/ncomms9038 PMC4569694 26333769

[B93] Sullivan B. J. Kipnis P. A. Carter B. M. Shao L.-R. Kadam S. D. (2021). Targeting ischemia-induced KCC2 hypofunction rescues refractory neonatal seizures and mitigates epileptogenesis in a mouse model. *Sci. Signal.* 14:eabg2648. 10.1126/scisignal.abg2648 34752143 PMC8763016

[B94] Talos D. M. Sun H. Kosaras B. Joseph A. Folkerth R. D. Poduri A. (2012). Altered inhibition in tuberous sclerosis and type IIb cortical dysplasia. *Ann. Neurol.* 71 539–551. 10.1002/ana.22696 22447678 PMC3334406

[B95] Tang B. L. (2020). The expanding therapeutic potential of neuronal KCC2. *Cells* 9:240. 10.3390/cells9010240 31963584 PMC7016893

[B96] Tang X. Drotar J. Li K. Clairmont C. D. Brumm A. S. Sullins A. J. (2019). Pharmacological enhancement of KCC2 gene expression exerts therapeutic effects on human Rett syndrome neurons and Mecp2 mutant mice. *Sci. Transl. Med.* 11:eaau0164. 10.1126/scitranslmed.aau0164 31366578 PMC8140401

[B97] Tang X. Kim J. Zhou L. Wengert E. Zhang L. Wu Z. (2016). KCC2 rescues functional deficits in human neurons derived from patients with Rett syndrome. *Proc. Natl. Acad. Sci. U.S.A.* 113 751–756. 10.1073/pnas.1524013113 26733678 PMC4725523

[B98] Tao R. Li C. Newburn E. N. Ye T. Lipska B. K. Herman M. M. (2012). Transcript-specific associations of SLC12A5 (KCC2) in Human prefrontal cortex with development, schizophrenia, and affective disorders. *J. Neurosci.* 32 5216–5222. 10.1523/JNEUROSCI.4626-11.2012 22496567 PMC3752043

[B99] Toda T. Ishida K. Kiyama H. Yamashita T. Lee S. (2014). Down-regulation of KCC2 expression and phosphorylation in motoneurons, and increases the number of in primary afferent projections to motoneurons in mice with post-stroke spasticity. *PLoS One* 9:e114328. 10.1371/journal.pone.0114328 25546454 PMC4278744

[B100] Tom B. Witko J. Lemay M. Singh A. (2018). Effects of bioengineered scaffold loaded with neurotrophins and locomotor training in restoring H-reflex responses after spinal cord injury. *Exp. Brain Res.* 236 3077–3084. 10.1007/s00221-018-5344-x 30132039

[B101] Tomita K. Kuwahara Y. Igarashi K. Kitanaka J. (2023). Therapeutic potential for KCC2-targeted neurological diseases ☆. *Jpn. Dent. Sci. Rev.* 59 431–438. 10.1016/j.jdsr.2023.11.001 38022385 PMC10665825

[B102] Tornberg J. Voikar V. Savilahti H. Rauvala H. Airaksinen M. S. (2005). Behavioural phenotypes of hypomorphic KCC2-deficient mice. *Eur. J. Neurosci.* 21 1327–1337. 10.1111/j.1460-9568.2005.03959.x 15813942

[B103] Verma V. Muddashetty R. Manjithaya R. Behnisch T. (2022). Pharmacological intervention in young adolescents rescues synaptic physiology and behavioural deficits in Syngap1+/- mice. *Exp. Brain Res.* 240 289–309. 10.1007/s00221-021-06254-x 34739555

[B104] Vyas P. Chaturvedi I. Hwang Y. Scafidi J. Kadam S. D. (2024). High doses of ANA12 improve phenobarbital efficacy in a model of neonatal post-ischemic seizures. *Int. J. Mol. Sci.* 25:1447. 10.3390/ijms25031447 38338726 PMC10855037

[B105] Woldman W. Cook M. J. Terry J. R. (2019). Evolving dynamic networks: an underlying mechanism of drug resistance in epilepsy? *Epilepsy Behav.* 94 264–268. 10.1016/j.yebeh.2019.03.003 30981121 PMC6581121

[B106] Woo N. S. Lu J. England R. McClellan R. Dufour S. Mount D. B. (2002). Hyperexcitability and epilepsy associated with disruption of the mouse neuronal-specific K-Cl cotransporter gene. *Hippocampus* 12 258–268. 10.1002/hipo.10014 12000122

[B107] Worthington A. Kalteniece A. Ferdousi M. D’Onofrio L. Dhage S. Azmi S. (2021b). Spinal Inhibitory dysfunction in patients with painful or painless diabetic neuropathy. *Diabetes Care* 44 1835–1841. 34385346 10.2337/dc20-2797

[B108] Worthington A. Kalteniece A. Ferdousi M. D’Onofrio L. Dhage S. Azmi S. (2021a). Optimal utility of h-reflex rdd as a biomarker of spinal disinhibition in painful and painless diabetic neuropathy. *Diagnostics* 11:1247. 10.3390/diagnostics11071247 34359330 PMC8306975

[B109] Yeo M. Chen Y. Jiang C. Chen G. Wang K. Chandra S. (2021). Repurposing cancer drugs identifies kenpaullone which ameliorates pathologic pain in preclinical models via normalization of inhibitory neurotransmission. *Nat. Commun.* 12:6208. 10.1038/s41467-021-26270-3 34707084 PMC8551327

[B110] Yeo M. Zhang Q. Ding L. Shen X. Chen Y. Liedtke W. (2022). Spinal cord dorsal horn sensory gate in preclinical models of chemotherapy-induced painful neuropathy and contact dermatitis chronic itch becomes less leaky with Kcc gene. *Front. Mol. Neurosci.* 15:911606. 10.3389/fnmol.2022.911606 36504679 PMC9731339

[B111] Zhou H. Y. Chen S. R. Byun H. S. Chen H. Li L. Han H. D. (2012). N-methyl-D-aspartate receptor- and calpain-mediated proteolytic cleavage of K+-Cl- cotransporter-2 impairs spinal chloride homeostasis in neuropathic pain. *J. Biol. Chem.* 287 33853–33864. 10.1074/jbc.M112.395830 22854961 PMC3460480

[B112] Zhou X. Zhu Y. Wang Z. Lin Z. Zhu D. Xie C. (2022). Rate-dependent depression: a predictor of the therapeutic efficacy in treating painful diabetic peripheral neuropathy. *Diabetes* 71 1272–1281. 10.2337/db21-0960 35234842

